# Trans-scaphoid lunate dislocation: A case series

**DOI:** 10.1016/j.radcr.2021.11.033

**Published:** 2021-12-15

**Authors:** Albert Gjeluci, Aleksandr Raskind, Bennett Dwan, Laith Yasin, Emad Allam

**Affiliations:** a Department of Radiology, Loyola University Medical Center, 2160 S 1st Ave, Maywood, IL 60153, USA; b Midwestern University Chicago College of Osteopathic Medicine, Downers Grove, IL, USA; c Loyola University Stritch School of Medicine, Maywood, IL, USA

**Keywords:** Lunate dislocation, Perilunate dislocation, Perilunate instability, Carpal instability, Mayfield classification, Avascular necrosis, ORIF, open reduction internal fixation, AVN, avascular necrosis, DRUJ, distal radioulnar joint, TFCC, triangular fibrocartilage complex

## Abstract

Trans-scaphoid lunate dislocation with volar displacement into the wrist/distal forearm is a devastating injury that most commonly occurs under situations of forceful impact to an extended wrist. Due to ligamentous disruption as well as fragile blood supply, these Mayfield type 4 injuries are associated with significant morbidity and long-term sequelae. Current treatment approaches to lunate dislocations depend on the severity and chronicity of the injury in addition to patient factors, with operative management potentially including ORIF or proximal row carpectomy. We report 5 cases of this rare injury pattern in 4 different patients.

## Background

Trans-scaphoid lunate dislocation is a very rare carpal injury that most commonly occurs in cases of high energy trauma to an outstretched hyperextended wrist. In such cases, there is typically volar dislocation/displacement of the lunate and scaphoid into the volar soft tissues of the wrist and forearm, with the severity of trauma often dictating how far these 2 carpal bones travel up the forearm. The relatively more common perilunate dislocation indicates disruption of the capitolunate articulation with dorsal dislocation of the capitate, whereas the relatively rare lunate dislocation indicates separation of the lunate from both the radius and the carpal bones with volar dislocation of the lunate through the space of Poirier. This injury pattern has been classically described using a 4-stage classification system originally formulated by Mayfield et al. [1]. Stage 1 begins with scaphoid rotation and disruption of the scapholunate ligament with stage 4 culminating in complete dislocation of the lunate and disruption of associated ligamentous structures [1]. Stage 4 injuries are associated with significant morbidity including increased risk of lunate AVN and complete carpal collapse [2]. Vascular supply to the lunate is maintained by palmar and dorsal vessels; complete dislocation can disrupt the tenuous blood supply leading to AVN [2]. In addition to vascular injury, median and ulnar nerve palsies have also been reported in the literature [3]. Long-term outcomes of these injuries include development of arthritis in up to 70% of these patients, with complex regional pain syndrome also playing a part in long-term morbidity [4]. Current standard of treatment for lunate dislocation is controversial, including ligament repair, ORIF to attempt to preserve wrist mobility [2], proximal row carpectomy to circumvent AVN and carpal instability, and wrist arthrodesis for chronic injuries. Earlier recognition and surgery for this injury have been associated with better prognosis [5].

We report a case series of 5 instances of trans-scaphoid lunate dislocation in 4 different patients. This is the largest case series of this entity to date with radiographic documentation.

## Case series

### Patient 1

A 41-year-old male presented after a motorcycle crash. The patient reported paresthesias in a right median nerve distribution. He was subsequently found to have a left pneumothorax requiring chest tube placement, left rib fractures, facial fractures, bilateral ankle fractures, left proximal ulnar fracture, and a right wrist deformity. Right wrist radiographs showed a trans-scaphoid lunate dislocation with severe volar displacement of the scaphoid fracture fragment and the lunate (Fig. 1a).

**Fig 1a:**
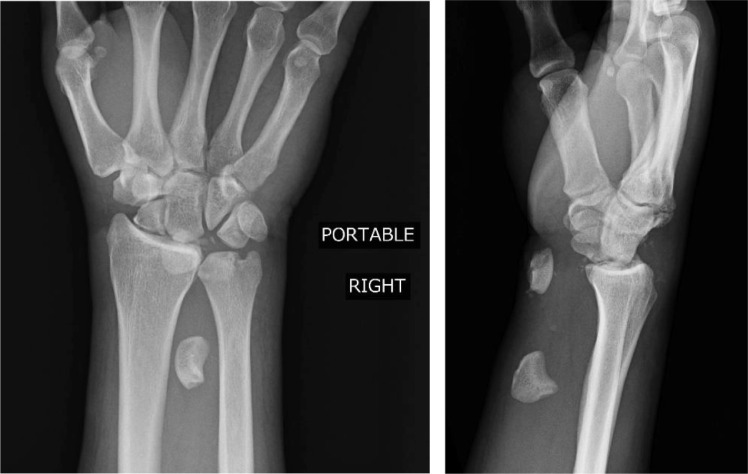
Patient 1: Initial radiographs of the right wrist showing a trans-scaphoid lunate dislocation. There is a fracture of the scaphoid waist with the proximal pole displaced into the volar wrist. There is severe dislocation of the lunate into the distal volar forearm.

On the fifth day post injury, the patient underwent right wrist surgery including proximal row carpectomy, carpal tunnel release, and excisional debridement of the forearm (Fig. 1b). Note that the patient was transferred to a hospital with specialists in hand surgery for this operation. On follow-up clinic visit 1 month later, the patient reported decreased pain and vastly improved numbness and tingling in his right hand.

**Fig 1b:**
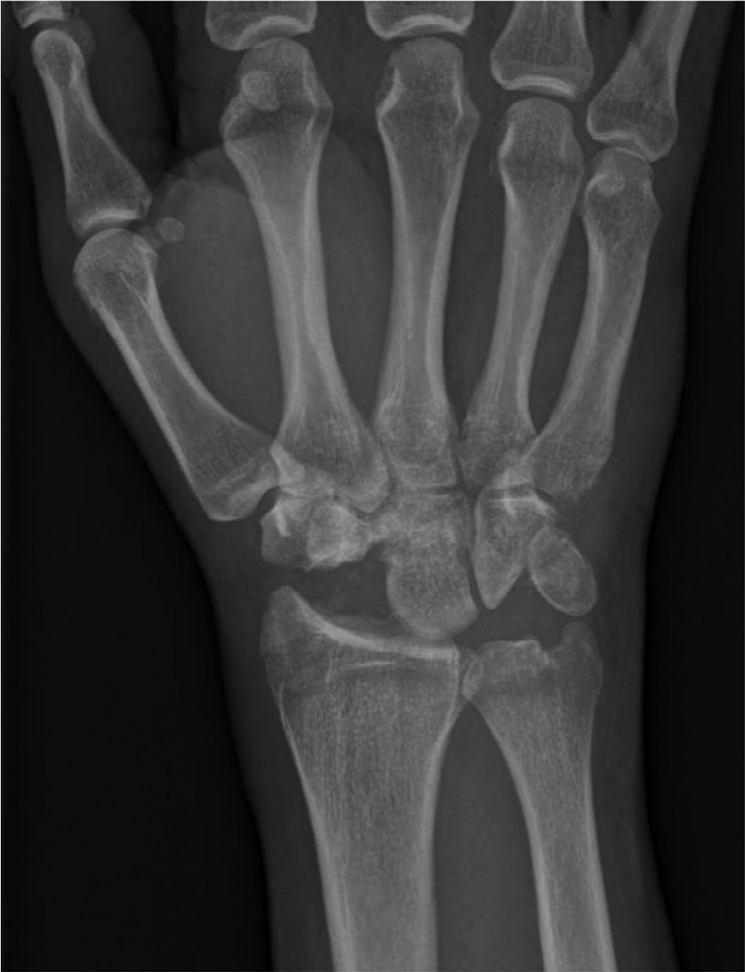
Patient 1: Postoperative radiograph of the right wrist showing changes of proximal row carpectomy.

### Patient 2

A 33-year-old male presented with multiple traumatic and burn injuries after jumping out from the third story window of a burning building. He had bilateral wrist deformities.

On the right, the patient had an elbow dislocation, distal radial diaphyseal fracture and distal radioulnar joint dislocation (Galeazzi type fracture-dislocation), and trans-capitate trans-scaphoid lunate dislocation (Fig. 2a). On the left, he had a distal radioulnar joint dislocation and trans-ulnar styloid trans-capitate trans-scaphoid lunate dislocation (Fig. 2b).

**Fig 2a:**
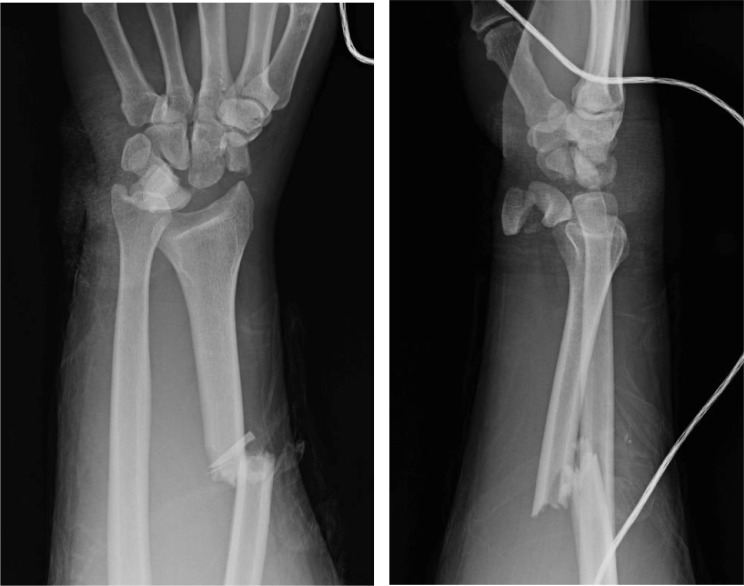
Patient 2: Initial radiographs of the right wrist demonstrate trans-capitate trans-scaphoid lunate dislocation as well as Galeazzi type fracture-dislocation including positive ulnar variance.

**Fig 2b:**
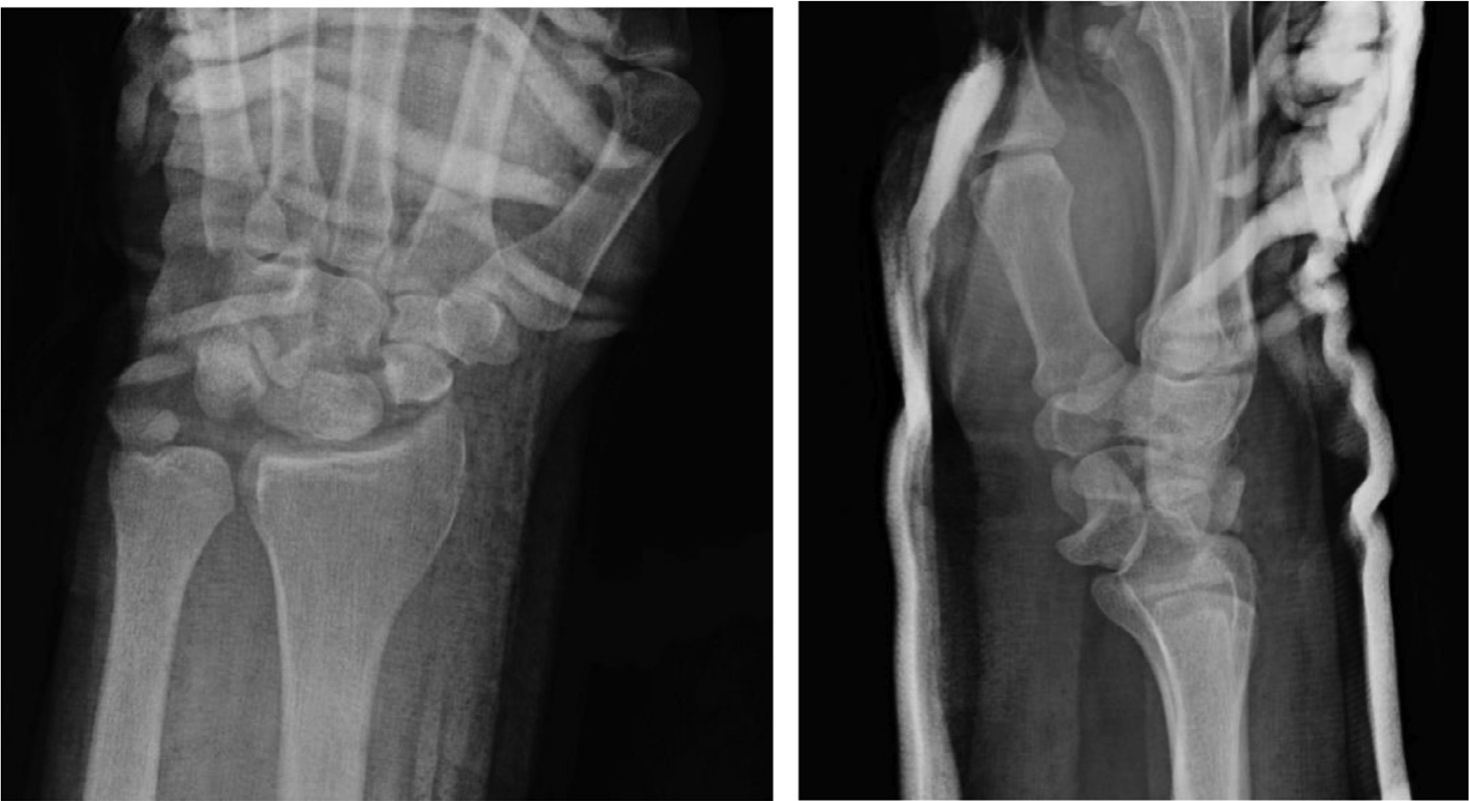
Patient 2: Initial radiographs of the left wrist demonstrate trans-ulnar styloid trans-capitate trans-scaphoid lunate dislocation. Overlying splint material is present.

The patient was admitted to the orthopedic service and received corrective surgeries on the second and third days of admission. The patient underwent ORIF of the right open radius fracture, right radial head arthroplasty, repair of right elbow collateral ligaments and capsule, and right volar forearm fasciotomy. He also underwent bilateral proximal row carpectomies, bilateral capitate screw fixation, bilateral dorsal spanning plate and screw fixation across the wrist, bilateral DRUJ pinning, bilateral TFCC repair, and debridement of the bilateral open wrist fractures (Fig. 2c).

**Fig 2c:**
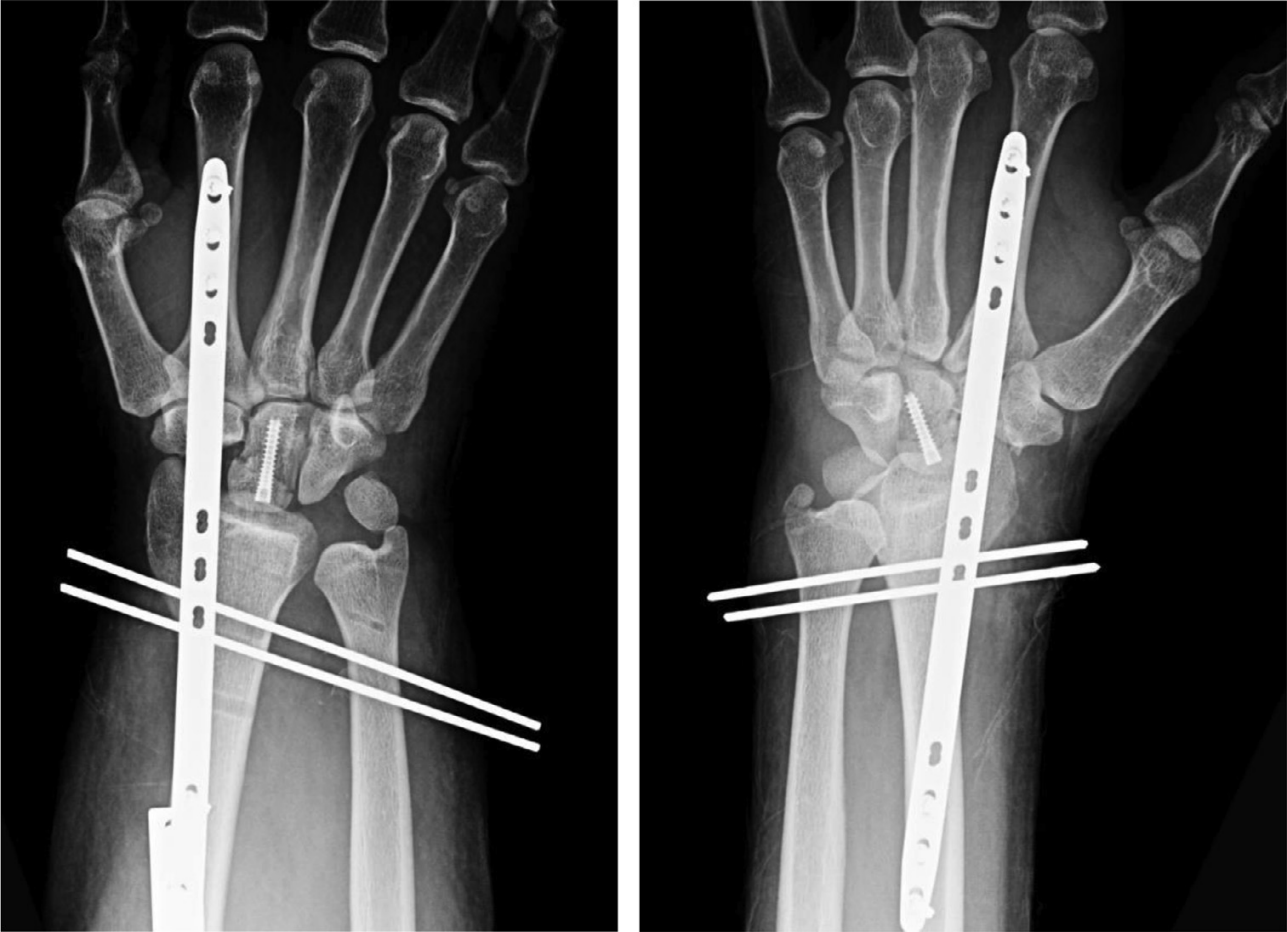
Patient 2: Postoperative radiographs of the bilateral wrists demonstrate proximal row carpectomy, fixation of the capitate with a headless screw, radiocarpal fixation with a long dorsal plate that spans the distal radius and index metacarpal, and percutaneous pinning of the DRUJ. Postoperative findings are similar bilaterally with the exception of partially imaged plate and screw fixation of the radial diaphysis on the right and a nondisplaced ulnar styloid fracture on the left.

The surgeries were successful and without any complications. The patient was discharged to a rehabilitation facility. On follow-up visits, he complained of residual pain, paresthesias, and limited range of motion in the right wrist. He subsequently suffered additional fractures of the right ulna and radius requiring further surgery. The worse outcome on the right was likely related to the more extensive and severe injuries he initially suffered on that side.

### Patient 3

A 19-year-old male presented after a high-speed motor vehicle crash that resulted in ejection. On arrival, he was intubated and became hemodynamically unstable, hypotensive and tachycardic in the radiology suite. He was found to have a right hemopneumothorax and an emergent thoracostomy with chest tube insertion was performed.

Initial physical exam and imaging revealed several skeletal injuries. In the left upper extremity, his injuries included a trans-ulnar styloid trans-triquetral trans-scaphoid lunate dislocation and third and fourth metacarpal base fractures (Fig. 3a).

**Fig 3a:**
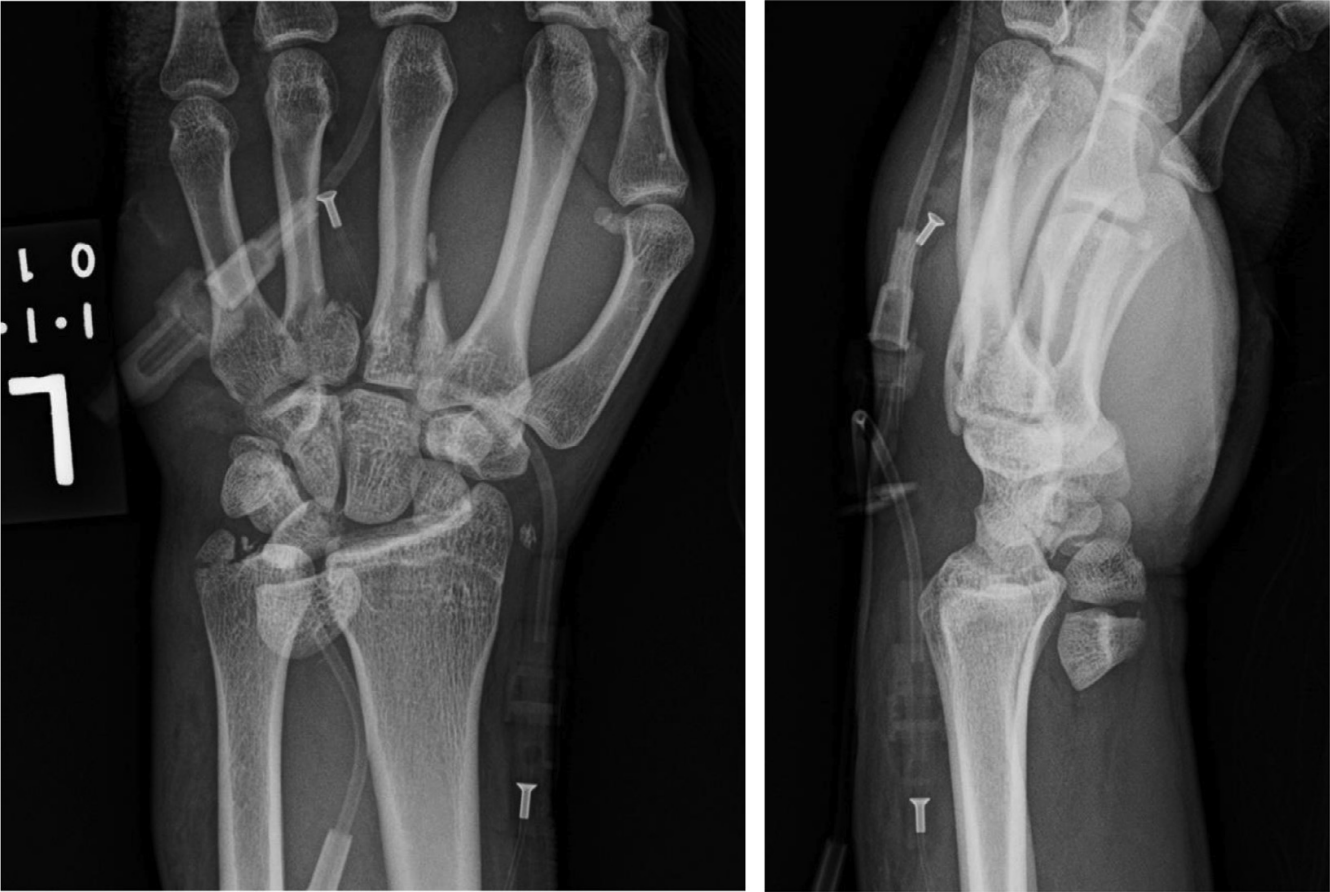
Patient 3: Initial radiographs of the left wrist demonstrating a trans-ulnar styloid trans-scaphoid lunate dislocation. A nondisplaced triquetral fracture was noted on subsequent CT. There are also fractures of the third and fourth metacarpal bases.

On day 3 of admission, the patient underwent pinning of the left carpal bones and the third and fourth metacarpal fractures, and screw fixation of the ulnar styloid fracture (Fig. 3b). A left middle finger laceration, 2 cm in length, was debrided and irrigated. A left middle finger extensor tendon zone 5 laceration was repaired, and he underwent left carpal tunnel release.

**Fig 3b:**
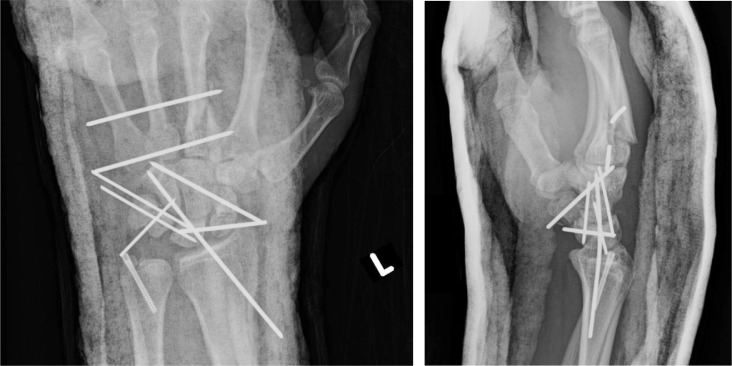
Patient 3: Postoperative radiographs of the left wrist demonstrating pinning across the carpal bones and the third and fourth metacarpal fractures, and screw fixation of the ulnar styloid fracture with improved alignment. Overlying splint material is present.

On follow-up visits, the patient reported significant left wrist weakness and loss of grip strength but minimal pain. Radiographs showed progressive collapse and resorption of the scaphoid and lunate, likely sequelae of AVN (Fig. 3c). Wrist fusion was discussed with the patient, but he was lost to follow-up.

**Fig 3c:**
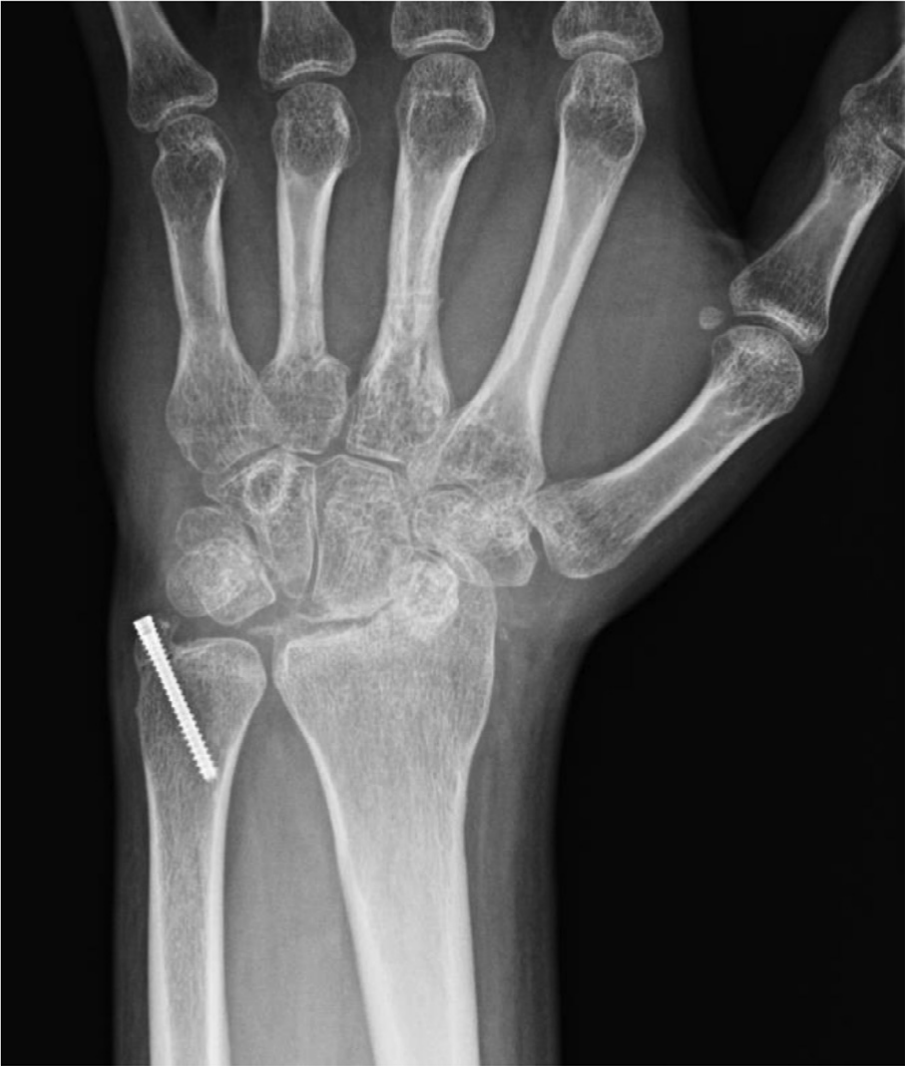
Patient 3: Subsequent radiographs obtained 7 months after the radiographs in Figure 3b show interval removal of hardware, with a headless screw remaining across the ulnar styloid fracture. The proximal pole of the scaphoid and the lunate are no longer visible, compatible with bony resorption/collapse; these bones were not surgically removed. The capitate now articulates with the distal radius with associated degenerative changes. The third and fourth metacarpal base fractures demonstrate interval healing.

### Patient 4

A 28-year-old male presented as a transfer from an outside hospital following a motorcycle crash. He reported pain in the right wrist.

Right wrist radiographs revealed a trans-radial styloid trans-scaphoid lunate dislocation (Fig. 4a). Closed reduction was performed and a thumb spica splint was placed (Fig. 4b). He was neurologically intact with a strong radial pulse. The patient was instructed to follow up with hand surgery but was lost to follow-up.

**Fig 4a:**
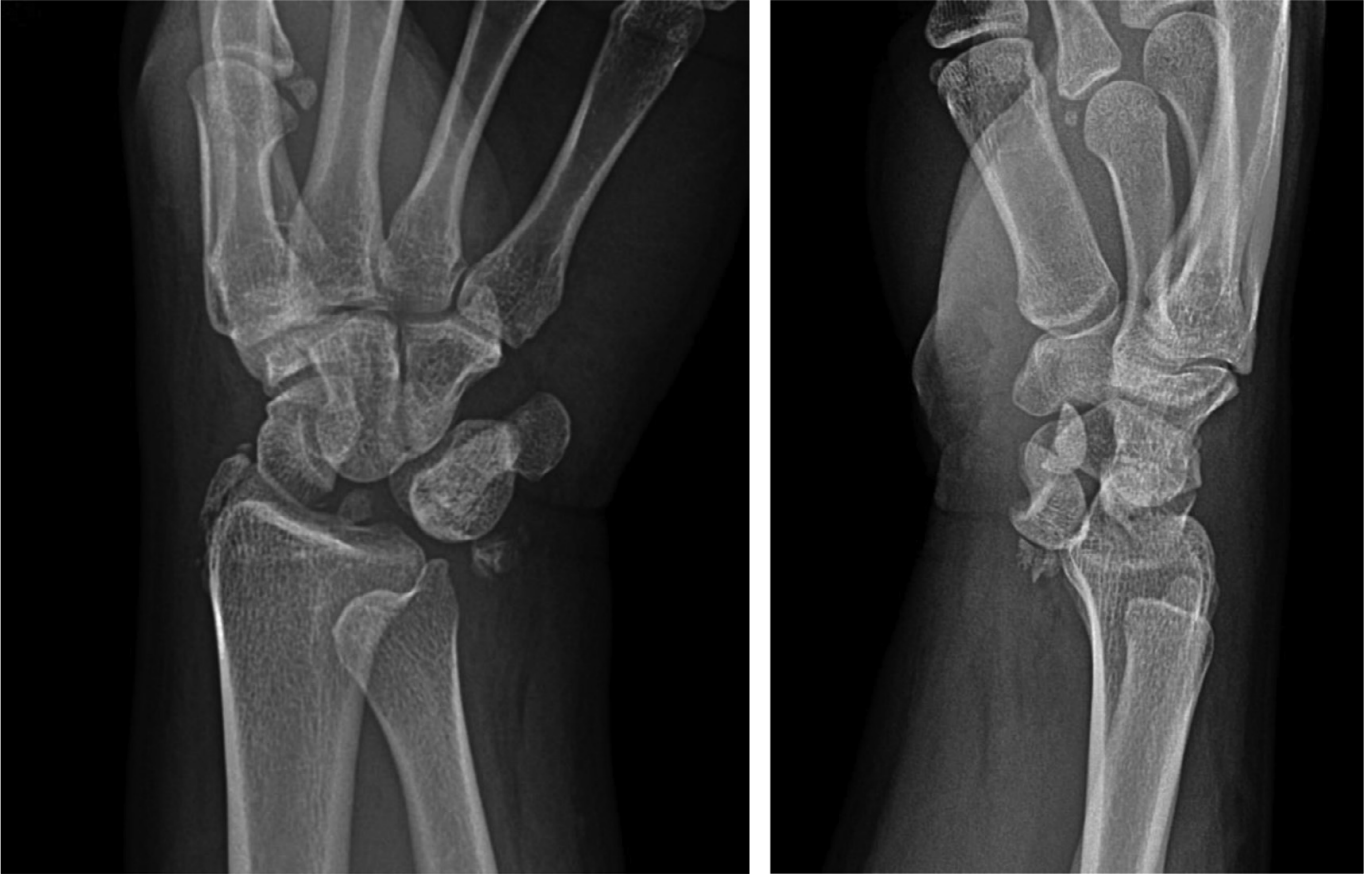
Patient 4: Initial radiographs of the right wrist reveal a trans-radial styloid trans-scaphoid lunate dislocation. Irregular fracture fragments at the ulnar volar aspect of the wrist may be from the scaphoid or pisiform. The radial styloid fracture is nondisplaced. There is negative ulnar variance.

**Fig 4b:**
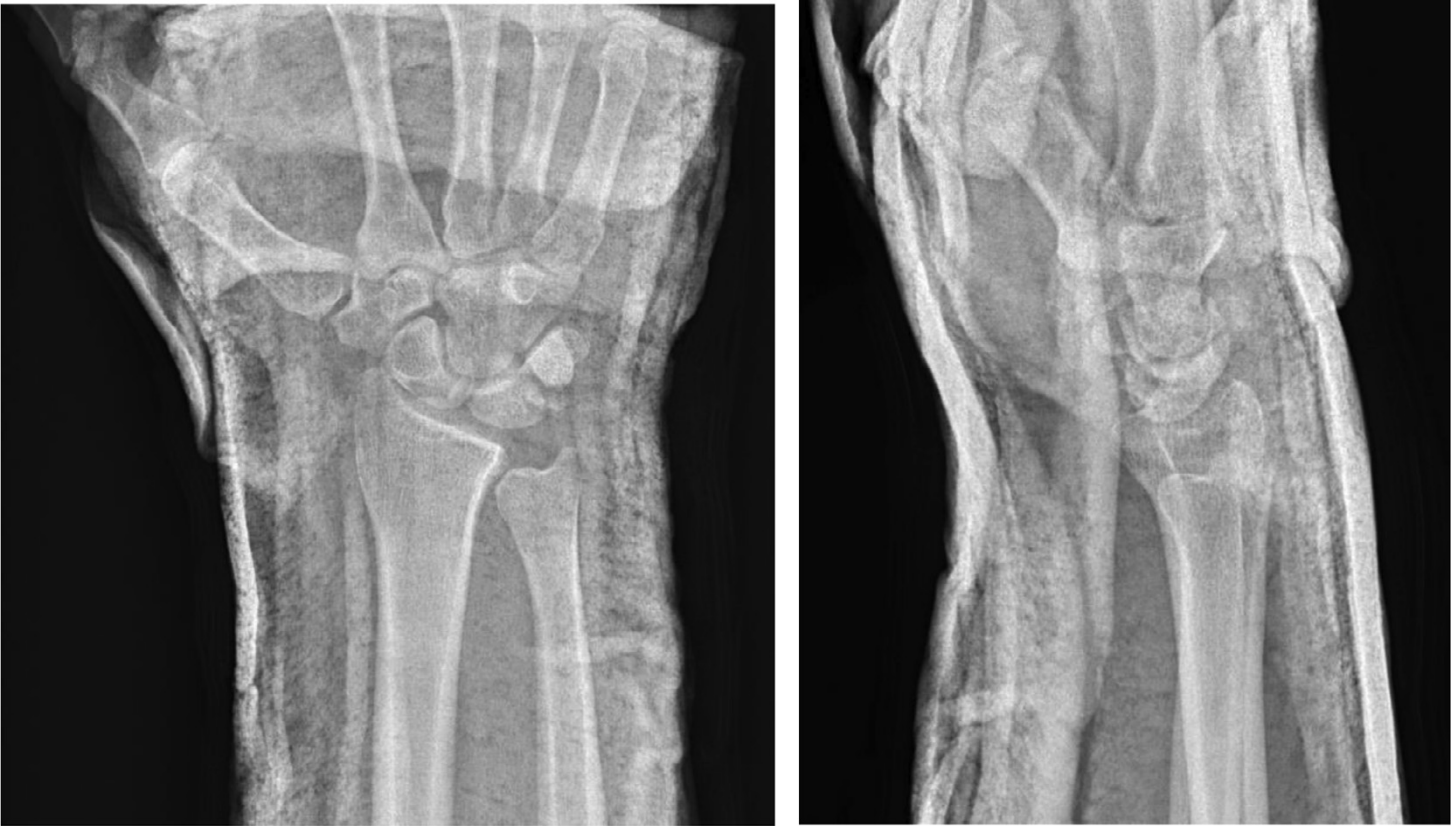
Patient 4: Subsequent radiographs of the right wrist following reduction and splinting demonstrating improved alignment.

## Discussion

Trans-scaphoid lunate dislocation is a very rare injury occurring mainly in high energy trauma where force is directed upon a hyperextended wrist. The most common mechanisms cited are falls, automobile accidents, and motorcycle crashes, all of which were seen in this case series. In Patient 1, the lunate not only dislocated volarly but also made its way into the distal portion of the forearm through the carpal tunnel. Volar dislocation of the lunate may cause nerve impingement and median nerve paresthesias. Stanbury et al. reported acute median nerve symptoms in about 24 to 45% of these patients, mentioning that swelling or contusion from trauma may play a role in symptoms [6]. They also reported that delayed onset of median nerve symptoms can occur due to edema and hemorrhage [6]. Marcuzzi et al. found that about 60% of perilunate injuries are associated with trans-scaphoid fractures [7]. Of these 60%, they found that about 72% of the scaphoid fractures occurred through the middle third [7]. All of our cases had trans-scaphoid lunate dislocations, with some demonstrating additional osseous injuries through the greater arc such as ulnar styloid, radial styloid, triquetral, and capitate fractures. Note that Patient 2 had bilateral trans-scaphoid lunate dislocations; this is the only such example in the published literature.

In terms of approaching these injuries, achieving the diagnosis in a prompt manner has been shown to be of utmost importance, and therefore radiologists play a critical role [8]. In a multicenter study evaluating 166 perilunate dislocations, open injuries and delayed treatment were negative prognostic factors for long-term clinical outcomes [8]. In both cases of early and delayed treatment, incidence of post-traumatic arthritis was high [8]. Despite these high rates of post-traumatic arthritis, many patients went on to have stable wrists and minimal pain [8]. Delays in diagnosis are not uncommon as patients with these injuries often have multiple other injuries that are more severe and life-threatening. When approaching complex wrist injuries such as perilunate or lunate dislocations, it is important to systematically evaluate the radiographs for associated greater arc fractures and to involve a hand surgeon in the patient's treatment plan.

## Patient consent

Formal consents are not required for the use of entirely anonymized images from which the individual cannot be identified - for example, x-rays, ultrasound images, pathology slides or laparoscopic images, provided that these do not contain any identifying marks and are not accompanied by text that might identify the individual concerned.
